# Synthesis of a novel ^89^Zr-labeled HER2 affibody and its application study in tumor PET imaging

**DOI:** 10.1186/s13550-020-00649-7

**Published:** 2020-06-03

**Authors:** Yuping Xu, Lizhen Wang, Donghui Pan, Junjie Yan, Xinyu Wang, Runlin Yang, Mingzhu Li, Yu Liu, Min Yang

**Affiliations:** 1 Key Laboratory of Nuclear Medicine, Ministry of Health, Jiangsu Key Laboratory of Molecular Nuclear Medicine, Jiangsu Institute of Nuclear Medicine, Wuxi, 214063 Jiangsu China; 2 Inner Mongolia Medical University, Hohhot, 010110 Inner Mongolia China; 3 Nanjing Medical University, Nanjing, 210029 Jiangsu China

**Keywords:** Human epidermal growth factor receptor-2, PET, Affibody, Zirconium-89

## Abstract

**Background:**

Human epidermal growth factor receptor-2 (HER2) is an essential biomarker for tumor treatment. Affibody is an ideal vector for preparing HER2 specific probes because of high affinity and rapid clearance from normal tissues, etc. Zirconium-89 is a PET imaging isotope with a long half-life and suitable for monitoring biological processes for more extended periods. In this study, a novel ^89^Zr-labeled HER2 affibody, [^89^Zr]Zr-DFO-MAL-Cys-MZHER2, was synthesized, and its imaging characters were also assessed.

**Results:**

The precursor, DFO-MAL-Cys-MZHER2, was obtained with a yield of nearly 50%. The radiochemical yield of [^89^Zr]Zr -DFO-MAL-Cys-MZHER2 was 90.2 ± 1.9%, and the radiochemical purity was higher than 95%. The total synthesis time was only 30 min. The probe was stable in PBS and serum. The tracer accumulated in HER2 overexpressing human ovarian cancer SKOV-3 cells. In vivo studies in mice bearing tumors showed that the probe was highly retained in SKOV-3 xenografts even for 48 h. The tumors were visualized with good contrast to normal tissues. ROI analysis revealed that the average uptake values in the tumor were greater than 5% IA/g during 48 h postinjection. On the contrary, the counterparts of MCF-7 tumors kept low levels ( ~ 1% IA/g). The outcome was consistent with the immunohistochemical analysis and ex vivo autoradiography. The probe quickly cleared from the normal organs except kidneys and mainly excreted through the urinary system.

**Conclusion:**

The novel HER2 affibody for PET imaging was easily prepared with satisfactory labeling yield and radiochemical purity. [^89^Zr]Zr-DFO-MAL-Cys-MZHER2 is a potential candidate for detecting HER2 expression. It may play specific roles in clinical cancer theranostics.

## Introduction

Targeting of a biomarker in cancers with specific agents is a promising strategy in the management of malignancies [1]. Human epidermal growth factor receptor type 2 (HER2) is a 185-kDa transmembrane protein and belongs to the family of receptor tyrosine kinases. It is involved in the signal transduction pathways regulating cell motility and proliferation. HER2 is an essential clinical tumor biomarker since it is overexpressed in various solid tumors, including ovarian, gastric, and breast cancers. Also, abundant expression of the receptor is associated with aggressiveness, recurrence, and reduced survival [2–4].

Monoclonal antibodies, including trastuzumab and pertuzumab have been used for therapy of HER2-positive cancers [5–7]. Current selection of patients for targeted therapy is mainly dependent on the status of HER2, determined by biopsy using immunohistochemistry or fluorescence in situ hybridization [8]. However, the invasive method may not be reliable because the HER2 expression is heterogeneous in the tumors and varies during the progress of the disease [9]. Almost 20% of the outcomes was inaccurate [10].

Noninvasive molecular imaging techniques such as single photon emission computed tomography (SPECT) and positron emission tomography (PET) provide a reliable method for repetitive investigating the distribution of the receptor in the whole body [11, 12]. Compared with the former, PET has higher image resolution and quality. PET scanner can sensitively detect gamma radiation from positron decay of nuclides (e.g., [^11^C] and [^18^F]). Due to high sensitivity, PET imaging with trace amount of radiotracers (10^−6−^10^−8^ grams) can accurately measure molecular targets in the living body without perturbing the biological system. PET with specific probes is a benefit for disease diagnosis and monitoring therapeutic response [13–15]. Radiolabeled antibodies have shown promise in identifying the presence of HER2 in the tumor [16–18]. For example, [^89^Zr]Zr-trastuzumab PET/CT detected unsuspected HER2-positive metastases in patients with HER2-negative primary breast cancer [19]. It also found lesions in patients with metastatic HER2-positive esophagogastric cancer [20]. However, the optimal images with favorable contrasts should be acquired at several days (3–5 days) after the administration of the antibodies [19].

Affibody is an engineered small protein that originated from the IgG-binding staphylococcal protein A and can be used as an alternative ligand towards HER2. It is an ideal compound for recognizing the desired targets due to ease of chemical synthesis, quick tumor accumulation, rapid blood clearance, etc. [21, 22]. ZHER_2:342_ affibody binds explicitly to HER2, and its derivatives have been labeled with PET nuclides ([^18^F], [^68^Ga], etc) [23–25]. ^18^F-labeled ZHER_2:342_ analog and [^18^F]F-FBEM–ZHER_2:342_ showed specific binding towards HER2-positive tumors and could be a benefit for detecting status of the receptor in response to therapeutic interventions [23, 26]. ^68^Ga-labeled HER2 affibody and ^68^Ga-ABY-025 discriminated HER2 positive and negative metastatic breast tumor in sixteen patients. After PET/CT scan with the tracer, treatment plans were changed in three patients [27, 28].

Besides the above short half-life radionuclides, few HER2 affibodies labeled with other PET isotopes are reported. Zirconium-89 is an attractive commercially available PET radionuclide with a long half-life (*T*_1/2_ = 78.4 h), which allows for imaging of biological processes at late time points [29]. Meanwhile, attachment of zirconium-89 to desferrioxamine (DFO) chelator coupled in bioactive substance (such as protein and peptides) can be achieved under mild conditions with excellent stability [30]. Besides, ^89^Zr-labeled compounds are also ideal surrogates for the corresponding therapeutic ^90^Y or ^177^Lu-labeled radiopharmaceuticals to calculate dosimetry and plan therapy program in preclinical or clinical studies [31].

Previous studies found that ^18^F or ^68^Ga-labeled modified HER2 affibody with a novel hydrophilic linker, [^18^F]FAl/[^68^Ga]Ga-NOTA-Cys-GGGRDN-ZHER_2:342_ (denoted as [^18^F]FAl/[^68^Ga]Ga-NOTA-Cys-MZHER2), owned satisfactory specific tumor uptakes and favorable tumor-to-muscle and tumor-to-blood ratios during 4 h after injection [32, 33]. Due to short half-life, further evaluating the properties of the affibody was difficult beyond 12 h. To better assess the characters of the modified HER2 affibody, the molecule was firstly coupled with a maleimide derivative of desferrioxamine, MAL-DFO, then the resulting compound was radiolabeled with zirconium-89. The efficiency of the resulting probe, [^89^Zr]Zr -DFO-MAL-Cys-MZHER2, was also investigated in tumor models.

## Methods

Cys-ZHER_2:342_ and Cys-MZHER2 were purchased from Apeptide Co., Ltd. (Shanghai, China). MAL-DFO was purchased from Macrocyclics (Dallas, TX). [^89^Zr]Zr-oxalate solution was supplied by Cyclotron VU(Netherlands). Human ovarian cancer cell lines SKOV-3 and breast cancer cell lines MCF-7 were obtained from Cell Bank of Shanghai Institutes for Biological Sciences. Female Balb/c nude mice were purchased from SLAC Laboratory Animal Co., Ltd., China. Analytic and preparative high-performance liquid chromatography (HPLC) were carried out according to the literatures [32, 33]. Radio thin-layer chromatography (TLC) was operated on silica gel impregnated glass fiber sheets and analyzed by a BioScan. Sodium citrate solutions (0.1 M) were used as solvent systems. Mass spectra were acquired from a Waters LC–MS system (Waters, Milford, MA).

### Affibody conjugation

**Fig. 1:**
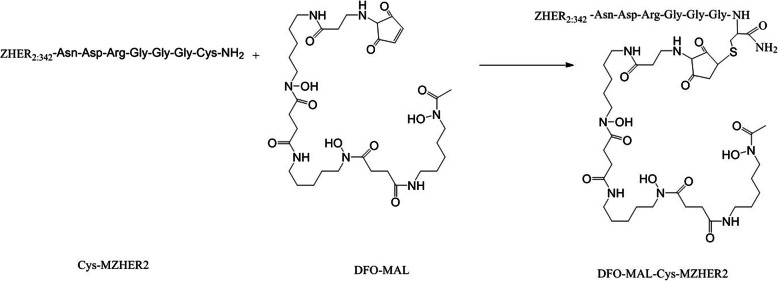
Scheme of synthesis of DFO-MAL-Cys-MZHER2

MAL-DFO (0.35 mg, 0.50 μmol) was dissolved in 2 ml ammonium acetate solution (2 M, pH = 7 ) and reacted with Cys-MZHER2 (3 mg, 0.40 μmol) at room temperature overnight (Fig. 1). The product was purified with preparative HPLC, followed by lyophilization, as previously described [32]. Mass spectrometry (MS) measured m/z 8077.5 for [MH]^+^ (C_351_H_560_N_104_O_111_S_2_, calculated molecular weight 8076.8).

### Preparing of ^89^Zr-DFO-MAL-Cys-MZHER2

**Fig. 2:**
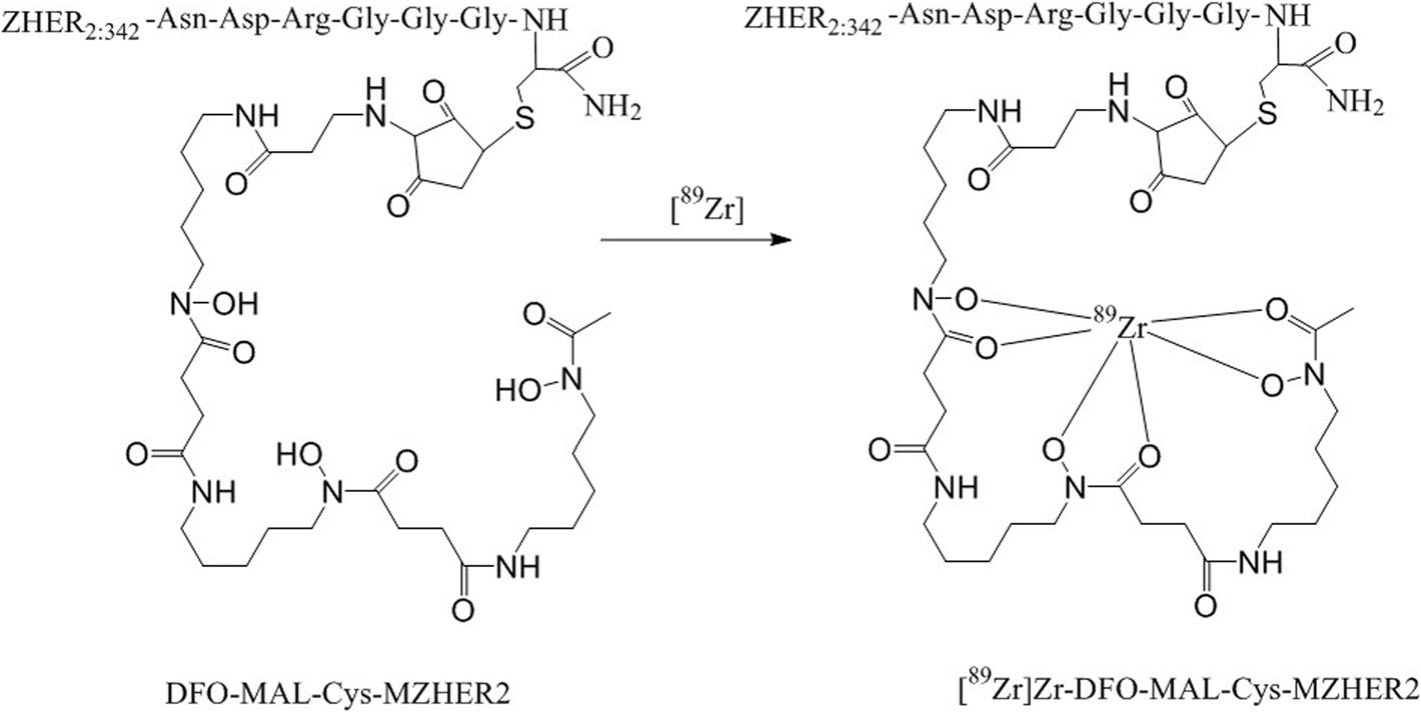
Scheme of radiosynthesis of [^89^Zr]Zr-DFO-MAL-Cys-MZHER2

DFO conjugated affibody, DFO-MAL-Cys-MZHER2 (200 μg, 25 nmol), was dissolved in 30 μL deionized water and incubated with 185 MBq [^89^Zr]Zr-oxalate in 1 mL 2 M Na_2_CO_3_ solution (pH = 4) for 20 min at room temperature (Fig. 2). After diluted with 10 mL deionized water, the complex was purified by a Varian BOND ELUT C18 column. After washing the column with 10 mL deionized water again, the product was eluted with 0.3 mL of 10 mM HCl in ethanol. The solution was diluted with 10 mL saline and passed through a 0.2- μm Millipore filter into a sterile vial. Radio HPLC and TLC were used for quality control.

### In vitro stability

Aliquots of [^89^Zr]Zr-DFO-MAL-Cys-MZHER2 solutions were incubated with 1 mL human serum or PBS for 48 h at 37 °C. At the preselected time points, the radiopurity was analyzed by TLC.

### Cell lines

Cells were cultured using RPMI-1640 medium supplemented with 10% (v/v) heat inactivated fetal bovine serum and grown as a monolayer at 37 °C in a humidified atmosphere containing 5% CO_2_.

### Cell uptake studies

Uptake studies of [^89^Zr]Zr-DFO-MAL-Cys-MZHER2 in SKOV-3 cells were performed according to the described method [32]. Cells (1 × 10^6^/well) were incubated at 37 °C for various times with 37 KBq-labeled affibody in a 0.5-mL serum-free DMEM medium. The nonspecific binding of the tracer was determined by co-incubation with 5 μM Cys-ZHER_2:342_. After washed with chilled PBS, the cell pellets in the tube were obtained by centrifugation and measured using a γ-counter (PerkinElmer). The cell uptake was expressed as the percentage of the added activity (%AA/10^6^ cells) after decay correction.

### Animal model

All animal experiments were performed according to the national guidelines and approved by the Ethics Committee of Jiangsu Institute of Nuclear Medicine. Tumor models were established by subcutaneously implanting 5 × 10^6^ SKOV-3 or MCF-7 tumor cells suspended in 0.2 mL PBS into the shoulder region of mice. When tumor sizes reached 100–300 mm^3^, the mice were used for the following experiments.

### MicroPET imaging

PET imaging was performed on a microPET scanner (Siemens Inc.). After anesthetized using isoflurane, the mice bearing tumors were placed in the center of the scanner and injected intravenously with 3.7 MBq [^89^Zr]Zr-DFO-MAL-Cys-MZHER2 in the presence or absence of excessive Cys-MZHER_2:342_ ( 10 mg/kg body weight) via the lateral tail vein. Static PET images of 10 min were performed at selected times after tracer injection. Quantitative analysis was operated using the reported methods [32, 33].

### Biodistribution

Mice were injected with 0.74 MBq of the tracer through the tail vein and sacrificed at 1, 4, 8, 18, 24, 48, and 72 h after administration, respectively. For the blocking study, four mice were coinjected with an excess of Cys-ZHER_2:342_ (10 mg/kg body weight) and killed at 1 h after administration. Tumor and normal tissues of interest were harvested and weighed. The radioactivity uptake in tissues was measured in the γ-counter and expressed as a percentage of the injected activity per gram of tissue (% IA/g).

### Autoradiography and histology

After microPET imaging, the tumors were harvested and sectioned into slices with 5 μm thickness at – 80 °C. Ex vivo autoradiography was conducted using the previous method [34]. To determine the intratumoral distribution of the tracer, the slices were placed on a phosphorimaging plate for 1 h. Phosphorimaging plates were read with a plate reader. Quantitative analysis was carried out using the OptiQuant software.

After radioactive decay, the slices were used for routine HE staining and HER2 analysis by immunohistochemistry. The procedures were processed following the published literature [33]. An epifluorescence microscope (Olympus, X81, Japan) was used to acquire the corresponding images.

### Statistical analysis

Statistical analyses were performed using GraphPad Prism. Data were analyzed using the unpaired, 2-tailed Student *t* test. Differences at the 95% confidence level (*p* < 0.05) were considered to be statistically significant.

## Results

### Chemistry

DFO conjugated affibody was readily prepared with a yield of 50%. The chemical purity of the compound was greater than 90% determined by analytical HPLC.

### Radiolabeling chemistry

**Fig. 3:**
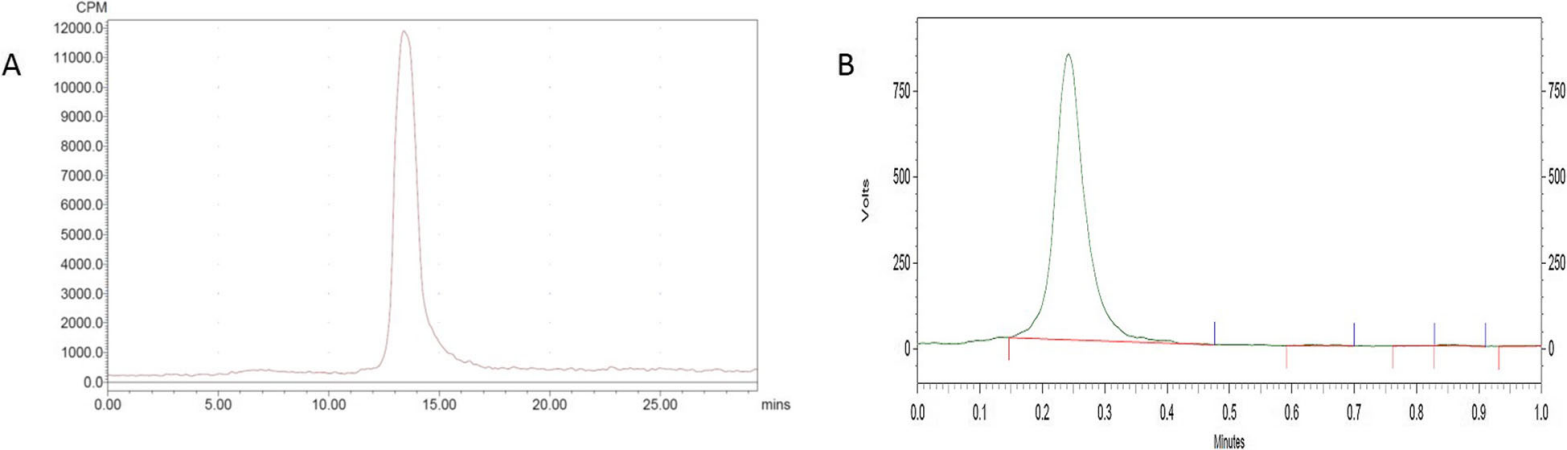
**a** HPLC chromatograms of [^89^Zr]Zr-DFO-MAL-Cys-MZHER2. **b** TLC chromatograms of [^89^Zr]Zr-DFO-MAL-Cys-MZHER2

The non-decay corrected radiochemical yield for [^89^Zr]Zr-DFO-MAL-Cys-MZHER2 was 90.2 ± 1.2%. Purification using C18 columns provided a radiochemical purity of more than 95%. HPLC analysis of the tracer showed one single peak with a retention time of 14 min (Fig. 3). [^89^Zr]Zr-DFO- MAL-Cys-MZHER2 showed a typical elution profile (Rf = 0.1–0.3) on radio TLC (Fig. 3). In comparison, free [^89^Zr]Zr-oxalate runs into the solvent front (Rf = 0.8–1.0) (Figure 1S).

### Stability studies in vitro

**Fig. 4:**
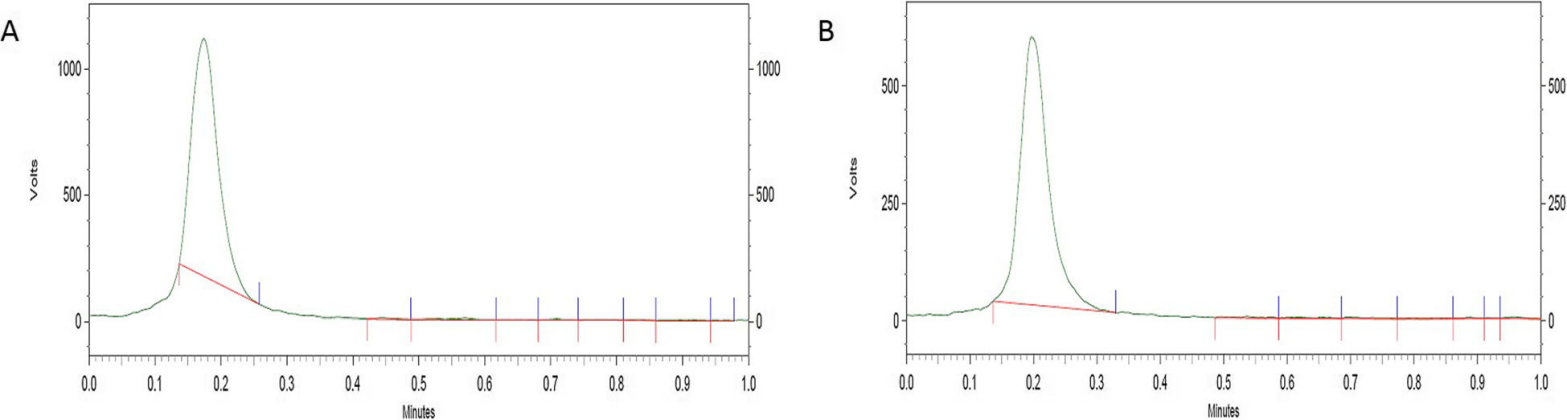
TLC chromatograms of [^89^Zr]Zr-DFO-MAL-Cys-MZHER2 in PBS (**a**) and serum (**b**) at 37 °C for 48 h, respectively

[^89^Zr]Zr-DFO-MAL-Cys-MZHER2 was stable during the investigated periods (Fig. 4). No free [^89^Zr]Zr-oxalate was found after incubation of the tracer in PBS or serum for 2 days at 37 °C.

### Cell uptake

**Fig. 5:**
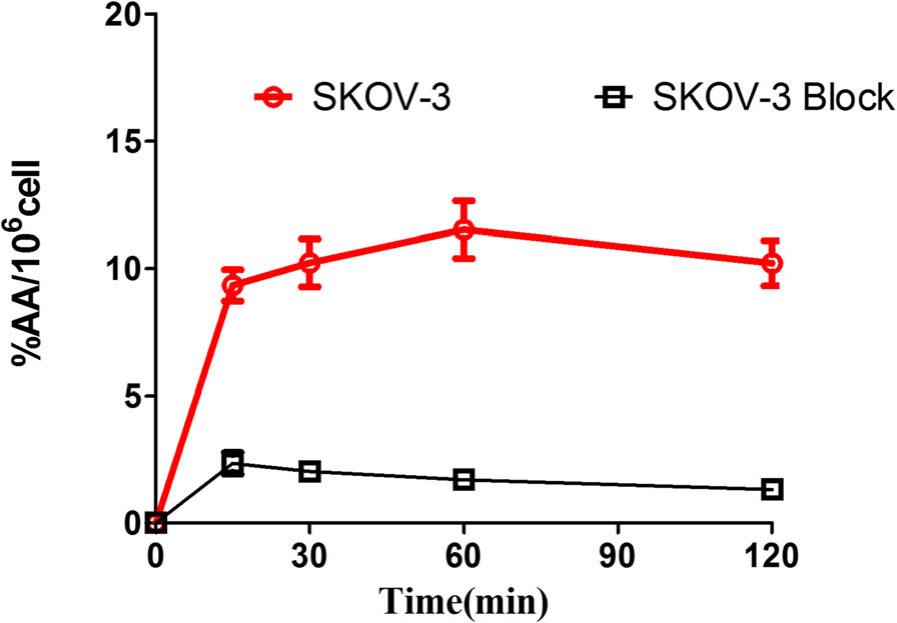
Cell uptake assay of [^89^Zr]Zr-DFO-MAL-Cys-MZHER2 in SKOV-3 cells

Cell uptake studies are shown in Fig. 5. The probe quickly accumulated in SKOV-3 and reached plateaus with 10.23 ± 0.94% AA/10^6^cells at 30 min incubation. By contrast, the uptake levels were significantly decreased in the presence of excess unlabeled Cys-ZHER_2:342_ at the same time points (2.35 ± 0.43%AA/10^6^cells).

### Small-animal PET imaging

**Fig. 6:**
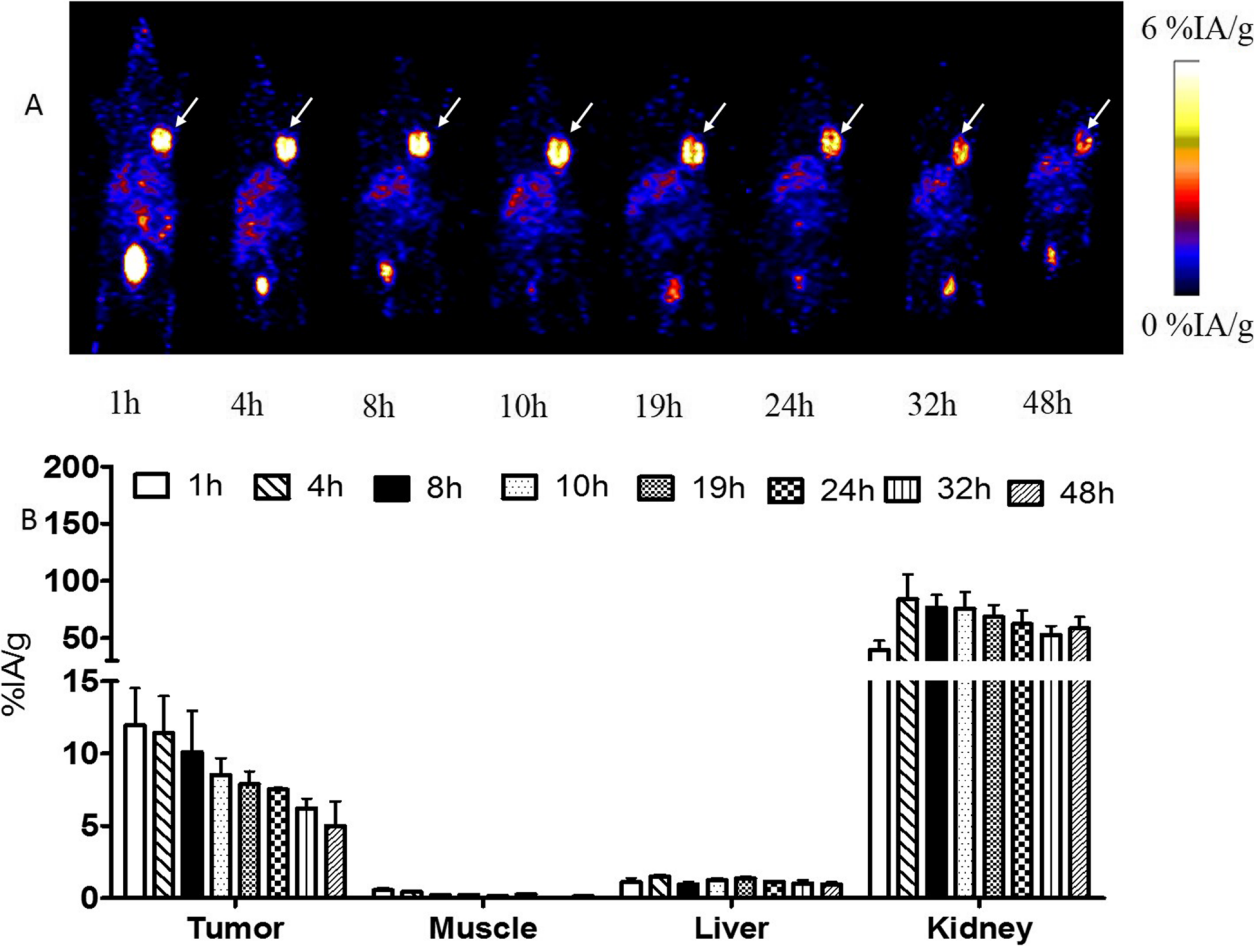
Decay-corrected whole-body PET images of mice bearing SKOV-3 tumors (**a**) after injection of [^89^Zr]Zr-DFO-MAL-Cys-MZHER2. **b** Quantification of radioactivities in SKOV-3 xenografts models. The tumors are indicated by the arrows

**Fig. 7:**
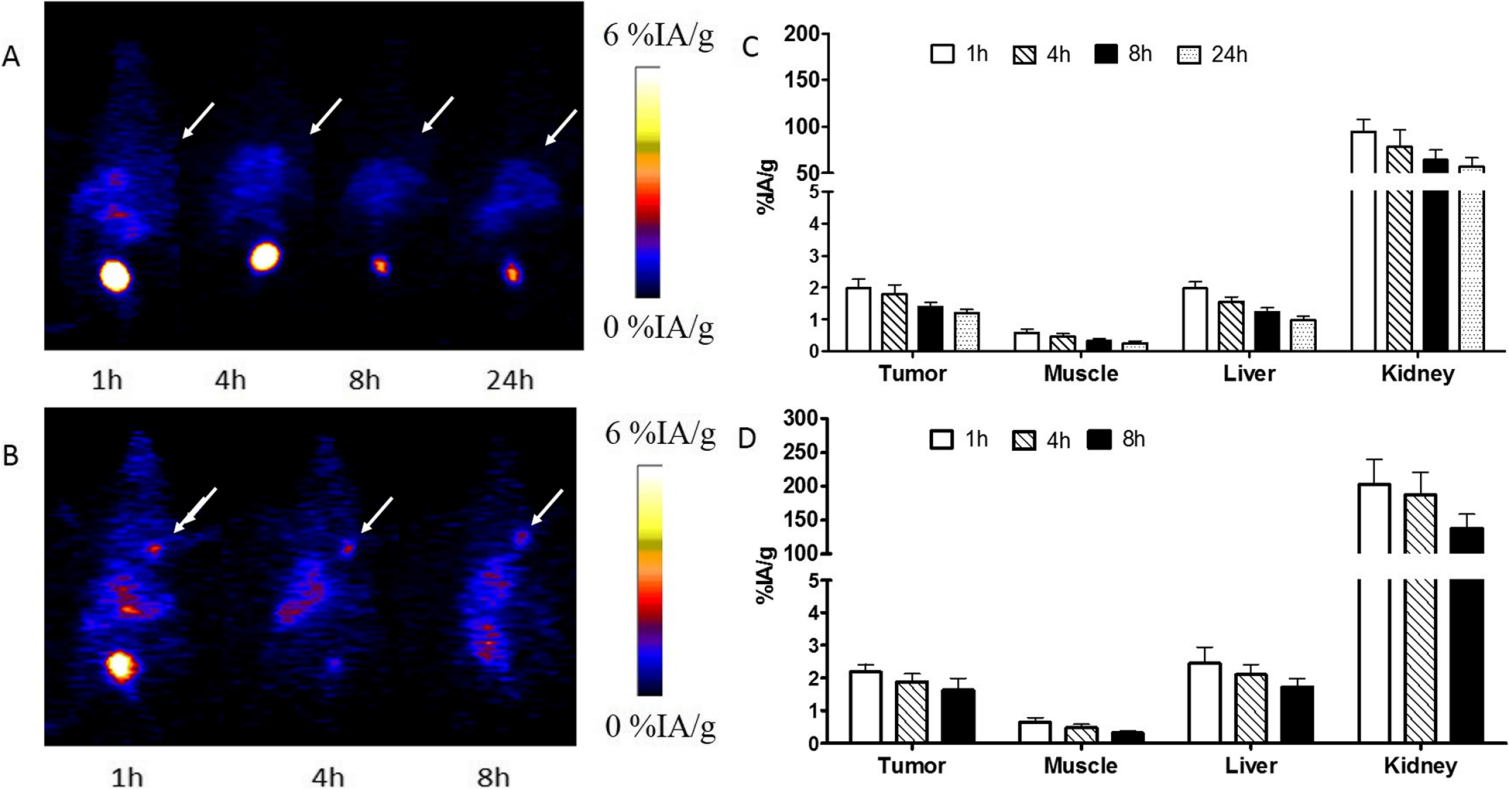
Decay-corrected whole-body PET images of mice bearing MCF-7 xenografts (**a**) after injection of [^89^Zr]Zr-DFO-MAL-Cys-MZHER2. **b** PET images of mice bearing SKOV-3 xenografts after injection of the tracer under block. **c** Quantification of [^89^Zr]Zr-DFO-MAL-Cys-MZHER2 in MCF-7 xenograft models and SKOV-3 xenograft models in the presence of excess block agents (**d**), respectively. The tumors are indicated by the arrows

MicroPET images of mice were listed in Figs. 6 and 7 and Figure 2S. SKOV-3 xenografts were clearly visualized with good contrast even after 48 h of administration. The SKOV-3 tumor uptakes of the tracer were 11.97 ± 2.52, 11.43 ± 2.51, 10.09 ± 2.83, 8.52 ± 1.15, 7.51 ± 0.39, and 4.99 ± 1.68% IA/g at 1, 4, 8, 10, 24, and 48 h after administration, respectively. In contrast, the radio signals in MCF-7 xenografts were weak. The MCF-7 tumor uptakes were 1.98 ± 0.28, 1.79 ± 0.29, 1.39 ± 0.14, and 1.21 ± 0.10% IA/g at 1, 4, 8, and 24 h after administration, respectively. Presaturation of HER2 in tumors by co-injection of nonlabeled Cys-MZHER_2:342_ caused a significant reduction of radioactivity accumulation in tumors (2.18 ± 0.23%IA/g at 60 min postinjection).

It also showed that uptake in the liver was deficient, and the highest values were nearly 2% IA/g at 1 h after injection. Accumulated radioactivities were found in kidneys. It suggested that the affibody is mostly excreted through the renal system and urinary tract.

### Biodistribution studies

**Fig. 8:**
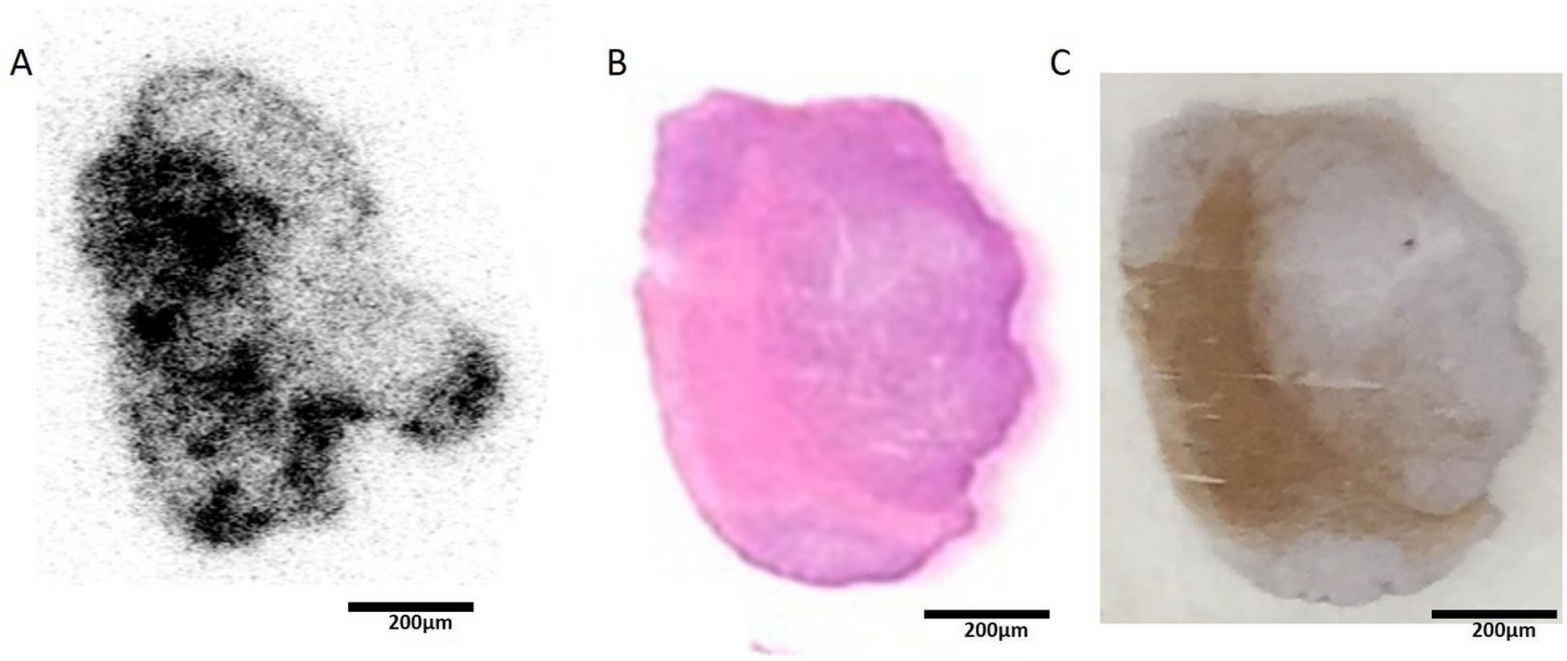
**a** Autoradiographic distribution of [^89^Zr]Zr-DFO-MAL-Cys-MZHER2 in tumor slices. HE staining (**b**) and immunohistochemical image (**c**) of HER2 in tumor slices

The biodistribution data of ^89^Zr-labeled affibody in mice bearing tumors are presented in Table 1. Similar with PET imaging, radioactivity concentration in SKOV-3 tumors was higher than those in MCF-7 tumors and other healthy organs except for kidneys. Acclamation in SKOV-3 tumors was 11.27 ± 1.55% IA/g at 1 h after administration and maintained 5.80 ± 0.75% IA/g at 48 h postinjection. [^89^Zr]Zr-DFO-MAL-Cys-MZHER2 uptake in the MCF-7 tumors was 1.76 ± 0.31% IA/g at 1 h postinjection. Under block with unlabeled HER2 affibody, the SKOV-3 tumor uptake of the tracer significantly reduced to 1.87 ± 0.15% IA/g at the same time point (Fig 8).

**Table 1:** Biodistribution of [^89^Zr]Zr-DFO-MAL-Cys-MZHER2 in mice bearing tumors at different time points after administration (*n* = 4 per group)

Tissues (%IA/g)	SKOV-3							SKOV-3 Block	MCF-7
	1 h	4 h	8 h	18 h	24 h	48 h	72 h	1 h	1 h
Blood	1.47 ± 0.47	0.52 ± 0.09	0.24 ± 0.10	0.16 ± 0.02	0.09 ± 0.01	0.04 ± 0.01	0.02 ± 0.00	1.03 ± 0.16	0.88 ± 0.19
Brain	0.73 ± 0.08	0.32 ± 0.06	0.28 ± 0.20	0.13 ± 0.03	0.10 ± 0.03	0.06 ± 0.01	0.03 ± 0.00	0.43 ± 0.05	0.34 ± 0.05
Heart	0.70 ± 0.24	0.55 ± 0.04	0.19 ± 0.08	0.17 ± 0.05	0.16 ± 0.07	0.10 ± 0.03	0.06 ± 0.01	0.62 ± 0.14	0.56 ± 0.06
Liver	2.03 ± 0.32	1.39 ± 0.38	0.63 ± 0.13	0.30 ± 0.06	0.17 ± 0.09	0.09 ± 0.03	0.05 ± 0.01	1.84 ± 0.39	1.65 ± 0.23
Spleen	1.47 ± 0.41	1.00 ± 0.27	0.68 ± 0.18	0.28 ± 0.09	0.20 ± 0.12	0.10 ± 0.03	0.06 ± 0.00	1.12 ± 0.10	0.92 ± 0.26
Lung	1.84 ± 0.50	1.27 ± 0.26	0.58 ± 0.14	0.35 ± 0.08	0.20 ± 0.05	0.10 ± 0.03	0.08 ± 0.01	0.68 ± 0.14	1.399 ± 0.14
Kidney	101.90 ± 11.31	145.33 ± 18.06	141.27 ± 6.21	146.57 ± 11.58	135.47 ± 6.71	128.47 ± 12.35	131.29 ± 20.36	175.83 ± 6.45	149.25 ± 15.52
Stomach	1.92 ± 0.31	1.19 ± 0.19	0.63 ± 0.22	0.36 ± 0.09	0.28 ± 0.07	0.17 ± 0.03	0.10 ± 0.02	1.16 ± 0.34	1.29 ± 0.12
Intestine	1.73 ± 0.27	1.16 ± 0.29	0.91 ± 0.43	0.57 ± 0.08	0.28 ± 0.04	0.13 ± 0.02	0.08 ± 0.01	1.89 ± 0.92	0.95 ± 0.11
Muscle	0.64 ± 0.12	0.44 ± 0.07	0.14 ± 0.02	0.09 ± 0.01	0.06 ± 0.01	0.03 ± 0.01	0.01 ± 0.00	0.58 ± 0.17	0.56 ± 0.08
Pancreas	1.49 ± 0.45	1.52 ± 0.28	0.82 ± 0.04	0.56 ± 0.05	0.38 ± 0.16	0.15 ± 0.03	0.06 ± 0.01	0.62 ± 0.14	1.38 ± 0.11
Bone	1.60 ± 0.33	1.36 ± 0.39	0.44 ± 0.02	0.36 ± 0.08	0.21 ± 0.10	0.15 ± 0.03	0.08 ± 0.02	0.71 ± 0.15	0.74 ± 0.10
Tumor	11.27 ± 1.55	10.95 ± 1.48	11.98 ± 1.24	9.82 ± 0.48	8.20 ± 0.82	5.80 ± 0.75	3.94 ± 0.21	2.18 ± 0.23	1.76 ± 0.31
Ratios									
Tumor/blood	8.38 ± 3.73	18.28 ± 2.10	43.09 ± 3.55	77.06 ± 14.67	89.00 ± 12.89	144.13 ± 17.93	198.00 ± 12.77	2.15 ± 0.26	1.98 ± 035
Tumor/muscle	17.80 ± 3.08	21.23 ± 2.09	79.52 ± 18.40	127.88 ± 12.16	138.07 ± 18.63	257.06 ± 29.95	393.50 ± 38.18	3.78 ± 1.02	3.37 ± 0.86

A rapid washout of radioactivity was noted from receptor-negative tissues except kidney. The uptake ratios of tumor-to-blood and tumor-to-muscle values increased from 8.38 ± 3.73 and 17.80 ± 3.08 at 1 h postinjection to 198.00 ± 12.77 and 393.50 ± 38.18 at 72 h postinjection in mice bearing SKOV-3 tumors, respectively.

### Ex vivo autoradiography and histology

Autoradiography studies showed that higher radioactivity accumulated in the periphery of tumors than those at internal tissues (Fig. 8). The ratios of radioactive intensity between the two regions were determined to be 5.03 ± 0.69.

The pathological analysis confirmed that the peripheral of tumor tissue grew vigorously, and the HER2 receptor was overexpressed. On the contrary, the internal tumor tissue grew slowly and even died with low levels of HER2. The results were consistent with the findings by autoradiography.

## Discussion

Desferrioxamine is a hexadentate chelator used for treating iron overload. It is consisted of three hydroxamate groups and forms a thermodynamically stable complex with zirconium [35]. For site-specific labeling, desferrioxamine-maleimide (MAL-DFO) was successfully introduced into MZHER2 by conjugated the thiol group in cysteine residue with the maleimide. Zirconium-89 was attached to DFO-MAL-Cys-MZHER2 under mild conditions with nearly quantitative yields. The radiochemical purity was satisfactory determined by both HPLC and TLC. Absence of radiolysis was detected in PBS and serum during in vitro incubation up to 48 h. The stability was similar with those of other [^89^Zr]Zr-labeled compounds. It means that ^89^Zr-labeled affibody could be prepared at least 1 day before preclinical or clinical PET studies.

Similar with ^18^F or ^68^Ga-labeled affibody, the ^89^Zr-labeled counterpart specifically binds to HER2-positive SKOV-3 tumor cells by in vitro cell uptake experiments. It meant that coupling the affibody with DFO-MAL could not significantly affect the performance of binding to the receptor.

To better understand the biology characters of the affibody, in vivo experiments including biodistribution studies and microPET imaging were firstly conducted in SKOV-3 and MCF-7 tumor models, which have been often applied for preclinical evaluating the specificity of radiolabeled HER2 affibody. It revealed that [^89^Zr]Zr-DFO-MAL-Cys-MZHER2 rapidly concentrated in SKOV-3 tumors after 1 h postinjection. The uptake values of [^89^Zr]Zr-DFO-MAL-Cys-MZHER2 in SKOV-3 tumors at 1 h postinjection (~ 11% IA/g) were similar to reported affibody in the HER2-positive xenografts. For example, the accumulated levels of [^18^F]F-FBEM-ZHER_2:342_, [^18^F]FAl-NOTA-MAL-MZHER_2:342_, [^68^Ga]Ga-ABY-002, [^68^Ga]Ga-NODAGA-ZHER_2:2395_, and [^68^Ga]Ga-DOTA-ZHER_2:2395_ in SKOV-3 xenografts at 1 h or 45 min postinjection were 9.73 ± 1.91, 18.60 ± 3.89, 7.81 ± 0.70, 15 ± 8, and 15 ± 2 %ID/g, respectively [26, 32, 36, 37]. Also, the corresponding uptake ratios of the tumor to background contrasts such as tumor-to-blood (8.38 ± 3.73) are also similar with those of [^18^F]F-FBEM-ZHER_2:342_ (7.5 ± 4.5), [^68^Ga]Ga-ABY-002 (6 ± 2), [^68^Ga]Ga -NODAGA-ZHER_2:2395_ (~ 8), and [^68^Ga]Ga-DOTA-ZHER_2:2395_ ( ~ 12), respectively [26, 32, 36, 37]. The differences in uptake values or tumor to contrast might be originated from the structure of different chelate agents coupled in the peptides. The uptake in SKOV-3 xenografts was about 10-fold higher than the counterparts of MCF-7 tumors at any time point. Compared with the blood pool and most of healthy tissues, the retention of the probe remained stable in SKOV-3 tumors over time. Even after 48 h postinjection, still, 40% of the initially accumulated radioactivity was observed in the SKOV-3 xenografts, and the image contrast was favorable. The uptake ratios of the tumor-to-blood and tumor-to-muscle after administration of [^89^Zr]Zr-DFO-MAL-Cys-MZHER2 at 72 h postinjection are nearly 200 and 400, respectively. The results are comparable to those of reported ^18^F or ^68^Ga-radiolabeled HER2 affibody at later time points (at most, 6 h) [26, 32, 36, 37]. It implied that the tracer might be a benefit for monitoring the status of HER2 in tumors with favorable contrast images for long periods at least 72 h.

The radioactivities distributing in the tumor showed significant heterogeneity by ex vivo immunohistochemistry and autoradiography. Abundant HER2 was expressed in the periphery of cancer ,and the corresponding radio signal was strong. On the contrary, weak radioactivity was detected in the internal necrotic tissues. Receptor specificity was also confirmed by decreasing the tumor uptake with excessive HER2 affibody. It implied that the targeting property of [^89^Zr]Zr-DFO-MAL-Cys-MZHER2 was consistent with the performances of ^18^F or ^68^Ga-labeled MZHER2 affibody [32, 33].

The uptake values in the liver are comparable to those of ^18^F or ^68^Ga-labeled affibody (~ 2% IA/g at 1 h p.i. then decrease to ~ 1% IA/g at 4 h p.i.). It suggested that the modification with a hydrophilic linker was effective to decline abdomen background. Similar to other ^89^Zr-labeled affibody such as [^89^Zr]Zr-DFO-ZEGFR_:2377_, higher radioactivity was found in the kidney after blocking. The detailed mechanism was not cleared. The scavenger receptor systems recycling proteins from the urines might mediate the re-absorptions of radiometal-labeled peptide in the kidney [38]. Despite this, high renal uptakes may not prevent the visualization of the tumor near the organ. For example, metastases in the adrenal gland were visualized after administration of ^111^In-labeled HER2 affibody, [^111^In]In-ABY-025 [39]. Low uptakes (< 2% IA/g) were observed in the bone. It suggested that the in vivo stability of [^89^Zr]Zr-DFO-MAL-Cys-MZHER2 was good since free ^89^Zr accumulates irreversibly in the mineralized bone.

## Conclusion

A novel PET tracer targeting HER2, [^89^Zr]Zr-DFO-MAL-Cys-MZHER2, was successfully prepared by radiolabeling modified affibody with zirconium-89. The preclinical study revealed that [^89^Zr]Zr-DFO-MAL-Cys-MZHER2 is a candidate radiotracer for specific, noninvasive detection of HER2 levels in tumors.

## Supplementary information

**Additional file 1: Figure S1**. TLC chromatograms of free [^89^Zr]Zr-oxalate

**Additional file 2: Figure S2**. Maximum intensity projects of mice bearing SKOV-3 (A), MCF-7 xenografts (B) after injection of [^89^Zr]Zr-DFO-MAL-Cys-MZHER2 without block and mice bearing SKOV-3 xenografts under block (C) respectively. The tumors are indicated by the arrows and circles.

## Data Availability

The data generated during the current study are available from the corresponding author on reasonable request.
