# Supplementary figures and images for: Synthesis of a novel 89Zr-labeled HER2 affibody and its application study in tumor PET imaging
Source: EJNMMI Res. 2020 Jun 3;10:58. doi: 10.1186/s13550-020-00649-7 (PMC7271293; doi:10.1186/s13550-020-00649-7)

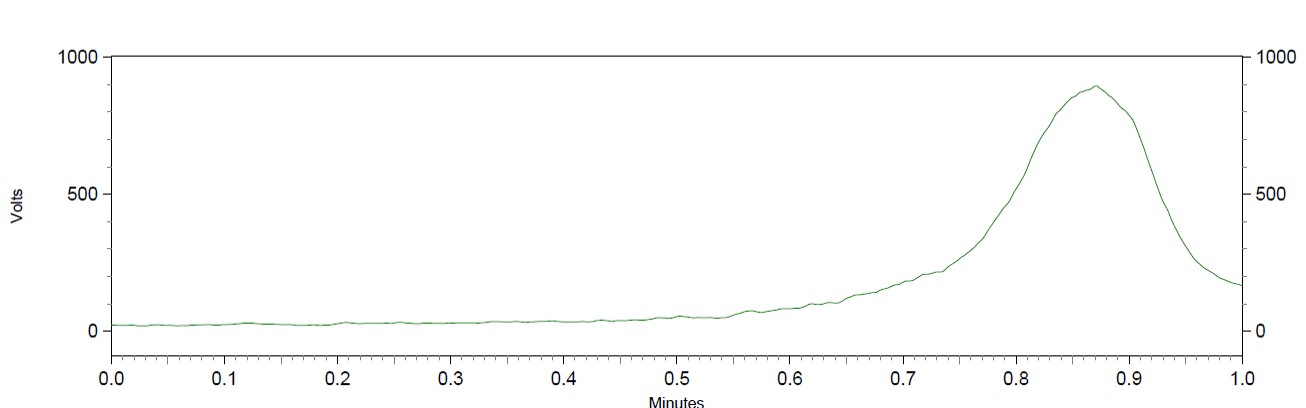

Supplement: Supplementary file 1 — Additional file 1: Figure S1. TLC chromatograms of free [89Zr]Zr-oxalate [file 13550_2020_649_MOESM1_ESM.jpg]

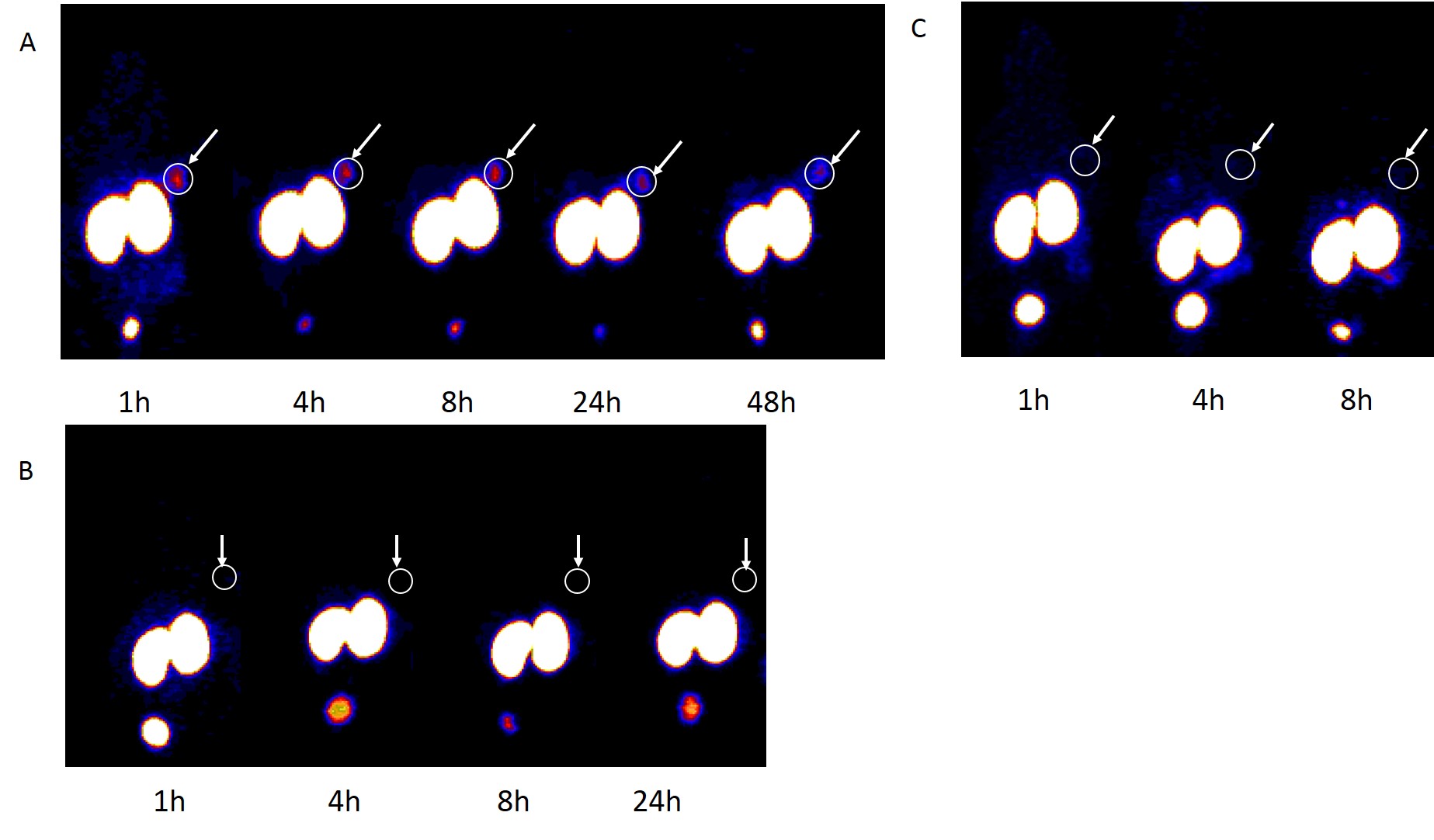

Supplement: Supplementary file 2 — Additional file 2: Figure S2. Maximum intensity projects of mice bearing SKOV-3 (A), MCF-7 xenografts (B) after injection of [89Zr]Zr-DFO-MAL-Cys-MZHER2 without block and mice bearing SKOV-3 xenografts under block (C) respectively. The tumors are indicated by the arrows and circles. [file 13550_2020_649_MOESM2_ESM.jpg]
